# Early postnatal care use by postpartum mothers in Mundri East County, South Sudan

**DOI:** 10.1186/s12913-017-2402-1

**Published:** 2017-06-26

**Authors:** Jonathan Izudi, Grace Denise Akwang, Dinah Amongin

**Affiliations:** 1 Institute of Public Health, International Health Sciences University, Box 7782, Kampala, Uganda; 2 Faculty of Health Sciences, Uganda Christian University, Box 4, Mukono, Uganda; 3 Department of Public Health, Faculty of Health Sciences, Busitema University, Box 1460, Mbale, Uganda

**Keywords:** Postnatal care, Postpartum care, Postpartum mothers, Maternal health, South Sudan

## Abstract

**Background:**

Globally, most maternal and newborn deaths are within the first week of delivery. Early postnatal-care (EPNC) visits between 2 and 7 days detects early morbidity and averts deaths. However, there is scarcity of information on use of EPNC in Mundri East County, South Sudan. This study investigated factors associated with EPNC use among postpartum mothers in Mundri East County, South Sudan.

**Methods:**

This was an analytical cross-sectional study of 385 postpartum mothers from 13 health facilities. Data was collected by structured questionnaires, entered in EpiData and analyzed with STATA at 5% significance level. Chi-squared, Fisher’s exact and Student’s t-tests were used for bivariate analysis and logistic regression for multivariable analysis.

**Results:**

The mean age of respondents was 27.9-years (standard deviation: 6.7), 276 (71.7%) were below 30-years, 163 (42.3%) were Muru ethnicity, 340 (88.3%) were single and 331 (86.1%) were unemployed. 44 (11.4%; 95% CI: 8.4–15.0) used EPNC. Poor health services access at government health facilities (Adjusted odds ratio (AOR) = 0.18; 95% CI: 0.05–0.61; *P* = 0.006), more than 1-h access to health facility (AOR = 0.27; 95% CI: 0.09–0.78; *P* = 0.015), at least secondary maternal education (AOR = 5.73; 95% CI: 1.14–28.74; *P* = 0.034) and receipt of PNC health education post-delivery (AOR = 3.47; 95% CI: 1.06–11.33; *P* = 0.004) were associated with EPNC use.

**Conclusions:**

Use of EPNC in Mundri East County, South Sudan was low. It was significantly reduced at government and inaccessible health facilities. However, it increased with receipt of PNC health education after delivery and at least secondary level of education.

## Background

High maternal and newborn deaths remain a pressing public health problem globally. Half of postnatal maternal [1, 2] and three-quarters of all newborn deaths [3] occur within the first 7-days after delivery. A maternal mortality trend analysis (1990–2015) by the World Health Organization (WHO) indicates that 830 women die annually from preventable causes related to pregnancy and childbirth [4]. In addition, the maternal mortality ratio (MMR), the number of maternal deaths per 100,000 live births was 12 in developed compared to 239 in developing countries [5]. Sub-Saharan Africa contributed 210,000 (more than half) deaths [5, 6] and 75% resulted from bleeding, postpartum sepsis, unsafe abortion, raised blood pressure and obstructed labor [4].

Use of postnatal care (PNC) services can drastically reduce these deaths through early identification of maternal and newborn danger signs [3, 7]. The four PNC visits recommended by WHO [8] are; (1) 24 h [2, 8, 9]; (2) 48–72 h [8]; (3) 7–14 days [8] and; (4) after 6 weeks [2, 7–9]. With the exception of few studies [10, 11], globally, many studies [12–15] focused on PNC at 6 weeks, a period when maternal and newborn deaths are lower compared to the first 7-days after delivery [16]. Such PNC studies are limited to survivors because the highest risk and proportion of maternal and newborn deaths is within the first 7-days post-delivery [16].

In South Sudan, the most recent WHO 1990–2015 data indicates that 789 mothers die in every 100,000 live births [5]. In spite of the high maternal deaths, PNC remains a rarely sought maternal and newborn health intervention.

Compared to other States in 2015, the Western Equatorial State (WES) of South Sudan had the highest MMR of 2327 per 100,000 live births (translating to over 170 maternal deaths per week) [17]. In the WES, three quarters of the maternal deaths were in Mundri East County but presently there is lack of data on early PNC (EPNC) visits, the use of PNC within 2–7 days by postpartum mothers. This study assessed the level and factors associated with EPNC use among postpartum mothers in Mundri East County, South Sudan.

## Methods

This study was conducted at 13 (one regional referral hospital, two county hospitals and 10 Primary Healthcare Centers (PHCC) purposively selected health facilities in Mundri East County, WES, South Sudan. In South Sudan, health services are provided at four different levels (central, state, county and community levels) each with a different diagnostic capacity and staffing requirements (including staff qualifications and responsibilities). Health services are further categorized as community care, primary health care, secondary care and specialized care. These various types of care are interlinked with a referral system [18]. Community Health Workers, Maternal and Child Health Workers and Home Health Promoters provide community healthcare at Primary Healthcare Units (PHCUs) and Primary Healthcare Centers (PHCCs) as main entry points. PHCUs provide the first level of interaction between the community and the formal health system and, provide basic preventive, promotive and curative care to about 15,000 people. In addition to services provided by PHCUs, PHCCs provide diagnostic/laboratory services and maternity care to an estimated 50,000 people.

County and State Hospital levels provide secondary, comprehensive-obstetric, in-patient and surgical care to 300,000 and 500,000 people respectively [18].

According to recent data, only 48% of pregnant women in WES attend antenatal care (ANC) visits: of these, 50.3% attended the fourth ANC visits and 17% attended four or more ANC visits. In terms of human resources for health, for every 100,000 people, only three health workers (Physicians, Nurses and Midwives) are available [19]. Consequently, only 10% of pregnant mothers deliver in the hands of skilled birth attendants (SBA) like Medical Doctors, Nurses and Midwives. Mundri East County has generally been peaceful until the period May 2015 and March 2016 when armed conflict erupted and led to displacement of over 1000 people. During the displacement, lack of essential medicines at health facilities constrained health service delivery and led to death of several people in Lozoh [20].

This study used a cross-sectional design to describe factors associated with EPNC use among postpartum mothers. We sampled postpartum mothers that had live births, were 15–49 years old, 8–14 days post-delivery and that attended PNC clinics (to receive immunization, contraception and growth monitoring services) between July 20, 2016 and September 18, 2016. Three hundred eighty five respondents based on Kish and Leslie’s formula [21] within a 95% confidence limit, 5% precision and 50% conservative estimation of EPNC use were included in the analysis. The number of participants interviewed at each health facility was obtained by dividing the sample size by the total number of sampled health facilities.

In each health facility, a systematic random sampling was used to establish a sampling interval by dividing the average number of postpartum mothers that attend PNC clinics by the required participant number. From the sampling interval, convenience sampling was used to select respondents. Between July and September 2016, trained and supervised Research Assistants collected data on use of PNC, socioeconomic and health services related factors by administering structured questionnaire. Interviews were conducted in quiet and conducive private room within the immunization clinic from Monday to Friday between 8.30 am-12.00 pm. All completed questionnaires were reviewed in real time for completeness and accuracy by the Research Team Lead.

The primary outcome, use of EPNC was defined as the proportion of postpartum mothers that had PNC visits within 2–7 days after delivery. Socioeconomic factors assessed included education levels measured as none, primary, secondary and beyond levels; occupation measured as non-employed, formal and self-employed; household income measured as estimated monthly total earnings in Sudanese pounds; marital status assessed as single (unmarried), married (monogamous) and separated (by divorce or death); household decision making measured as who makes final decision regarding use of maternal and child health services.

Health services variables assessed included level of health facility measured as PHCU, PHCC or hospital; health facility ownership measured as government or PNFP (private not for profit); use of ANC measured as the number and history of ANC visits in the last pregnancy; health education on PNC during ANC visit measured as having ever received PNC message in the last pregnancy; place of delivery measured as delivery in a health facility or a non-health facility setting (at home or on the way to a health facility); mode of delivery in the last pregnancy measured as spontaneous vaginal delivery (SVD), caesarean section or assisted delivery; SBA measured as last delivery by a Medical Doctor, Nurse or Midwife; being informed of PNC visits measured as reception of PNC messages (focused on benefits and schedules) at the time of discharge by a SBA; knowledge of postpartum complications measured as knowing some maternal postpartum complications (bleeding, offensive vaginal discharge, fever and severe abdominal pain among others) and some newborn complications (reddening of and pus discharge from the umbilical cord, restlessness, poor suckling and convulsions and so forth); time to reach the nearest health facility was taken as more or less than 1-h of reach and distance was calculated as less or more than 5-km of reach; presence of healthcare providers at the health facility was taken as having at least a health worker on duty at any time of the day and week.

Data was double entered in EpiData version 3.1 (EpiData Association, Odense, Denmark) [22], checked for data quality and exported to STATA Version 12 (StataCorp, College Station, TX, USA) for univariate, bivariate and multivariate analysis using a 5% significance level.

Frequencies and percentages were calculated for categorical variables, and measures of central tendency for continuous variables. Tests of associations were conducted using chi-squared test when the cell size was equal to or above five, Fisher’s exact test when the cell count was less than five, and Student’s t-test for continuous outcomes. Significant variables were considered for logistic regression analysis and examined by odds ratios (OR) with corresponding 95% confidence interval (CI) and probability values (*P*-values).

Ethical approval was obtained from the Institutional Review Boards of Uganda Christian University (Mukono Campus), the County Health Department and Lui Hospital, South Sudan. All data collection tools were forward and backward translated and pretested outside the study area before data collection. All participants gave written or thumb printed informed consent.

## Results

### Socio-demographic characteristics of respondents

The mean age of the 385 respondents was 27.9 years (Standard deviation (SD): 6.7 years) and median age was 27.0 years (Interquartile range (IQR):23–32 years). 276 (71.9%; 95% CI: 66.9–76.1) were below 30-years old, 163 (42.3%; 95% CI: 37.3–47.4) were Muru ethnic tribe, 340 (88.3%; 95% CI: 84.7–91.3) were not married, 331 (86.0%; 95% CI: 82.1–89.3) had no employment and 221 (57.4%; 95% CI: 52.3–62.4) had delivered their first child (Table 1).

**Table 1:** Use of EPNC, East Mundri County

Variable	Used EPNC?		Total (Percentage, 95% CI
	No	Yes	
	No. (percentage)	No. (percentage)	
Health facility grade			
Primary Healthcare Unit	221 (91.0)	22 (9.0)	243 (63.1, 58.1–67.9)
Primary Healthcare Centre and Hospital	120 (84.5)	22 (15.5)	142 (36.9; 32.1–41.9
Health facility ownership			
Private not for profit (PNFP)	59 (76.6)	18 (23.4)	77 (20.0; 16.1–24.3)
Government	282 (23.4)	26 (8.4)	308 (80.0; 75.7–83.9)
Age of respondents			
Less or equals 30	242 (87.7)	34 (12.3)	276 (71.9; 66.9–76.1)
More than 30	99 (90.8)	10 (9.2)	109 (28.3; 23.9–33.1)
Mean/SD	28.0 ± 6.7	26.8 ± 6.1	385
Religion of respondents			
Catholic	139 (90.9)	14 (9.1)	153 (39.7; 34.8–44.8)
Moslem	16 (76.2)	5 (23.8)	21 (5.4; 3.4–8.2)
Anglican	186 (88.2)	25 (11.8)	211 (54.8; 49.7–59.9)
Tribe of respondents			
Muru	141 (41.4)	22 (50.0)	163 (42.3; 37.3–47.4)
Mundu	15 (4.4)	3 (6.8)	18 (4.7; 2.8–7.3)
Balanda	9 (2.6)	4 (9.1)	13 (3.4; 1.8–5.7)
Baka	40 (11.7)	4 (9.1)	44 (11.4; 8.4–15.0)
Bari	38 (11.1)	7 (15.9)	45 (11.7; 8.6–15.3)
Dinka	29 (8.5)	1 (2.3)	30 (7.8; 5.3–10.9)
Mundari	59 (20.2)	3 (6.8)	72 (18.7; 14.9–23.0)
Parity (children ever born)			
One	193 (87.3)	28 (12.7)	221 (57.4; 52.3–62.4)
Two and more	148 (90.2)	16 (9.8)	164 (42.6; 37.6–47.7)
Maternal highest educational level			
None	179 (93.7)	12 (6.3)	191 (49.6; 44.5–54.7)
Primary	134 (86.5)	21 (13.5)	155 (35.3–45.3)
Secondary and over	28 (71.8)	11 (28.2)	39 (10.1; 7.3–13.6)
Maternal occupation			
None	298 (90.0)	33 (10.0)	331 (86.0; 82.1–89.3)
Employed	38 (80.9)	9 (19.1)	47 (12.2; 9.1–16.0)
Self-employed	5 (71.4)	2 (28.6)	7 (1.8; 0.7–3.7)
Marital status			
Single	307 (90.3)	33 (9.7)	340 (88.3; 84.7–91.3)
Married	26 (83.9)	5 (16.1)	31 (8.1; 5.5–11.2)
Separated	8 (57.1)	6 (42.9)	14 (3.6; 2.0–6.0)
Spouse’s highest educational level			
None	181 (92.4)	15 (7.6)	196 (50.9; 45.8–56.0)
Primary	91 (84.3)	17 (15.7)	108 (28.1; 23.6–32.8)
Secondary and over	69 (85.2)	12 (18.2)	81 (21.0; 17.1–25.5)

### Use of EPNC

**Fig. 1:**
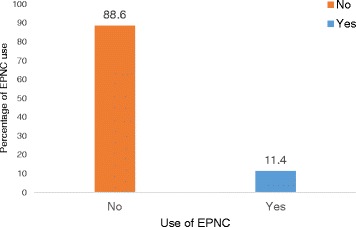
Percentage of EPNC use by postpartum mothers

Overall, 44 (11.4%; 95% CI: 8.4–15.0) respondents used EPNC (Figure 1). Of respondents that used EPNC, 22 (9.0%) were from PHCUs while the other 22 (15.5%) were from PHCCs and hospitals, 18 (23.4%) from PNFP health facilities while 26 (8.4%) were from government health facilities, 34 (12.3%) were below 30-years, 5 (23.8%) were Muslim, 28 (12.7%) had only a child, 11 (28.2) had reached secondary education or beyond, 9 (19.1) had formal employment, 6 (42.9) had separated/divorced and 12 (18.2) had spouses’ that reached secondary education or beyond (Table 1).

### Univariable analysis of factors associated with use of EPNC

Respondents that accessed health services at government health facilities were less likely to use early PNC compared to those from PNFP health facilities (UOR = 0.30, 95%CI: 0.16–0.59); *P* < 0.001). Respondents with primary (UOR = 2.34, 95% CI: 1.11–4.92); *P* = 0.025) and secondary education or beyond (UOR = 5.86, 95CI: 2.36–14.56); *P* < 0.001) had increased EPNC use compared to those without any formal education. Being married (UOR = 1.79, 95%CI: 0.64–4.97; *P* = 0.265) was not statistically significantly associated with increased use of EPNC compared to single respondents. Also respondents that had separated (UOR = 6.98, 95% CI: 2.28–21.33, *P* = 0.001) had statistically significantly increased EPNC use compared to single respondents (Table 2).

**Table 2:** Multivariable analysis of factors associated with early postnatal care in East Mundri County, South Sudan

Variable	Used early PNC?		Univariable logistic regression analysis		Multivariable logistic regression analysis for all variables with *p* < 0.05 at univariable logistic regression analysis	
	No	Yes	UOR (95% CI)	*P*-value	AOR (95% CI)	*P*-value
	No. (%)	No. (%)				
Health facility ownership						
Private not for profit (PNFP)	59 (76.6)	18 (23.4)	1	-	1	-
Government	282 (23.4)	26 (8.4)	0.30 (0.16–0.59)	<0.001	0.18 (0.05–0.61)	**0.006**
Mothers educational level						
None	179 (93.7)	12 (6.3)	1	-	1	-
Primary	134 (86.5)	21 (13.5)	2.34 (1.11–4.92)	0.025	2.57 (0.78–8.48)	0.122
Secondary and over	28 (71.8)	11 (28.2)	5.86 (2.36–14.56)	<0.001	5.73 (1.14–28.74)	**0.034**
Marital status						
Single	307 (90.3)	33 (9.7)	1	-	1	-
Married	26 (83.9)	5 (16.1)	1.79 (0.64–4.97)	0.265	0.62 (0.10–3.80)	0.609
Separated	8 (57.1)	6 (42.9)	6.98 (2.28–21.33)	0.001	2.89 (0.52–16.20)	0.227
Attended ANC in last pregnancy						
No	145 (96.0)	6 (4.0)	1	-	1	-
Yes	196 (83.8)	38 (16.2)	4.69 (1.93–11.38)	0.001	1	-
Had four or more ANC visits						
No	173 (85.6)	29 (14.4)	1	-	1	-
Yes	23 (71.9)	9 (28.1)	2.33 (0.98–5.54)	0.055	1.24 (0.29–5.34)	0.770
Educated on PNC visits at ANC						
No	173 (85.6)	29 (14.4)	1	-	1	-
Yes	23 (71.9)	9 (28.1)	2.59 (1.09–6.20)	0.033	0.69 (0.15–3.22)	0.639
Place of delivery						
Health facility	121 (78.1)	34 (21.9)	1	-	1	-
Home	220 (95.7)	10 (4.3)	0.16 (0.08–0.34)	<0.001	0.30 (0.04–2.28)	0.245
Mode of delivery						
Spontaneous vaginal	302 (90.7)	31 (9.3)	1	-	1	-
Caesarean section	12 (54.6)	10 (45.5)	8.12 (3.24–20.31)	<0.001	2.35 (0.56–9.86)	0.244
Forceps	27 (90.0)	3 (10.0)	1.08 (0.31–3.77)	0.901	0.24 (0.03–1.76)	0.160
Skilled birth attendance						
No	236 (95.6)	11 (4.5)	1	-	1	-
Yes	105 (76.1)	33 (23.9)	6.74 (3.28–13.85)	<0.001	0.70 (0.10–4.83)	0.714
Informed of PNC visits after delivery						
No	295 (95.5)	14 (4.5)	1	-	1	-
Yes	46 (60.5)	30 (39.5)	13.74 (6.78–27.85)	<0.001	3.47 (1.06–11.33)	**0.004**
Knows postpartum complications						
No	198 (95.2)	10 (4.8)	1	-	1	-
Yes	143 (80.8)	34 (19.2)	4.71 (2.25–9.84)	<0.001	1.19 (0.32–4.46)	0.799
More than 1-h to reach nearest health facility						
No	86 (76.1)	27 (23.9)	1	-	1	-
Yes	254 (93.7)	17 (6.3)	0.21 (0.11–0.41)	<0.001	0.27 (0.09–0.78)	**0.015**
Health providers were friendly and caring						
No	187 (95.9)	8 (4.1)	1		1	
Yes	150 (80.7)	36 (19.4)	5.61 (2.53–12.43)	<0.001	3.39 (0.08–11.40)	0.050
Health providers ever present at PNC clinic						
No	251 (94.0)	16 (16.0)	1	-	1	-
Yes	90 (76.3)	28 (23.7)	4.86 (2.51–9.40)	<0.001	1.75 (0.58–5.27)	0.318

Percentages were calculated as row percentages, n/N; *UOR* Unadjusted odds ratio, *AOR* Adjusted odds ratio, *CI* Confidence interval

In terms of ANC visits, respondents that attended ANC visits compared to those that never were statistically significantly more likely to use EPNC (UOR = 4.69; 95% CI: 1.93–11.38; *P* = 0.001).

At least four ANC visits compared to less than four visits (UOR = 2.33; 95% CI: 0.98–5.54; *P* = 0.055) was not significantly associated with use of EPNC. Respondents that had health education on PNC visits compared to those that had no health education on PNC had increased EPNC use (UOR = 2.59, 95% CI: 1.08–6.20; *P* = 0.033).

10 (4.3%) respondents that delivered at home compared to 34 (21.9%) that delivered in a health facility used EPNC. Home delivery was associated with reduced EPNC use compared to health facility delivery (UOR = 0.16; 95% CI: 0.08–0.34; *P* < 0.001). Also, compared to respondents that had SVD, caesarean section delivery (UOR = 8.12; 95% CI: 3.24–20.31; *P* < 0.001) and assisted vaginal delivery (UOR = 1.08; 95% CI: 0.31–3.77; *P* = 0.901) was associated with increased EPNC use.

SBA by a Midwife, Nurse or a Medical Doctor compared to non-SBA was associated with increased EPNC use (UOR = 6.74; 95% CI: 3.28–13.85; *P* < 0.001). Respondents that had been informed of PNC checkups after delivery were more likely use EPNC compared to those that had not been informed of PNC visits after delivery (UOR = 13.74; 95% CI: 6.78–27.85; *P* < 0.001).

Respondents that knew postpartum complications had increased EPNC use compared to those that never knew postpartum complications (UOR = 4.71; 95% CI: 2.25–9.84; *P* < 0.001). Respondents that reported more than 1-h to reach the nearest health facility compared to those that reported less than 1-h had reduced EPNC use (UOR = 0.21; 95% CI: 0.11–0.41; *P* < 0.001) (Table 2).

Respondents that lived over 5-Kilometers from the nearest health facility for PNC had decreased EPNC use compared to those that lived less than 5-km (UOR = 0.21; 95% CI: 0.08–0.54; *P* = 0.001). Whenever health workers were friendly, EPNC use statistically significantly increased in contrast to when they are reportedly unfriendly (UOR = 5.61; 95% CI: 2.53–12.43; *P* < 0.001). Reported presence of health workers at health facilities was statistically significantly associated with increased EPNC use compared to reported absence (UOR = 4.86; 95% CI: 2.51–9.40; *P* < 0.001).

### Multivariable analysis of factors associated with use of EPNC

After adjusting for health facility ownership, mothers educational level, marital status, history of and number of ANC visits in last pregnancy, history of health education on PNC during ANC visits, place and mode of delivery, skilled birth attendance, ever receiving information on PNC visits after delivery from a SBA, knowledge of postpartum complications, estimated time taken to reach the nearest health facility, health providers interpersonal relations with mothers and their availability at the health facility, there was a statistically significantly reduced use of EPNC among participants that accessed health services at government health facilities (AOR = 0.18; 95% CI: 0.05–0.61; *P* = 0.006) and that took more than 1-h to reach the nearest health facility (AOR = 0.27; 95% CI: 0.09–0.78; *P* = 0.015). Conversely, use of EPNC statistically significantly increased among respondents that received information on when to return for PNC visits after delivery from a SBA (AOR = 3.47; 95% CI: 1.06–11.33; *P* = 0.004) and that had secondary level of education or beyond (AOR = 5.73; 95% CI: 1.14–28.74; *P* = 0.034) (Table 2).

## Discussion

The present study investigated EPNC use by postpartum mothers in Mundri East County, South-Sudan. 11.4% of postpartum mothers used EPNC. This implies that a substantial proportion of postpartum mothers do not use EPNC in Mundri East County. Previously PNC was regarded as a neglected [2], poorly used [23], inadequately recognized and a weak reproductive, maternal and child health intervention [24]. In reports from the literature, low rates of PNC use was reported elsewhere [25–27]. Interestingly, the proportion of EPNC use in Mundri East county was even much lower than 15.4% reported in Eastern Uganda [10]. Our results emphasis the need to strengthen use of EPNC by recognizing and addressing barriers to utilization.

Postpartum mothers that accessed health services at government health facilities had reduced EPNC use compared to those from PNFP health facilities. This confirms results from past published study on EPNC use in Eastern Uganda that found significantly reduced use of EPNC among postpartum mothers at government than private health facilities [10]. Perceived differences in quality of care between PNFP and government health facilities among postpartum mothers might account for the increased use of EPNC at PNFP health facilities. In Palestine [25] and Brazil [28], past studies indicated increased PNC use at private than public health facilities. A study that compared client satisfaction with maternal health services between government and private hospitals in Jos, Nigeria found clients at private hospitals were more satisfied than those at government health facilities [29].

Maternal educational level (secondary education or beyond) increased EPNC use. This is in conformity with previous studies [10, 30]. Maternal education increase the ability to take action regarding health and disease. So mothers with formal education have better control over the determinants of health compared to illiterate mothers (or those without formal education). A past study indicates that low maternal or paternal literacy levels hinder use of maternal health services [13].

We found mothers that received information on PNC visits after delivery (before health facility discharge) had increased use of EPNC. This confirms the importance of health education in enhancing and sustaining use of available health services. This result concurs with earlier studies in Uganda [10, 15]. In particular, the provision of PNC health education to postpartum mothers before health facility discharge increased EPNC use by almost 10-fold in Eastern Uganda [10]. Our findings has implications for research and practice. In practice, we suggest the inclusion of PNC health education by SBAs at the time of discharge of postpartum mothers from health facilities. In research, there is need to conduct an interventional study to evaluate the effect of point of discharge PNC health education on use of EPNC.

We found postpartum mothers that accessed the nearest health facility after 1-h had reduced use of EPNC. When health facilities are distant, access to routine maternal health services and emergency care is reduced due to relatively high costs in paying for motorized transport system (when available) and lengthy travel time.

In most African countries, difficulties in accessing health facilities remain a big hindrance to use of available health services [15, 26]. To increase use of EPNC, health facilities must be within five kilometers radius. In South Sudan, only 44% of the population are settled within five kilometers radius to a functional health facility [19]. Secondly, the protracted civil war led to total destruction of the entire social sector (including but not limited to the health sector). South-Sudan is thus still recovering from the devastating civil war effects. The consequences will hence continue to affect health service delivery for years. However, our results highlight the importance of improving access to health services (preferably within 5-km radius) to avert maternal and newborn deaths.

This study concedes several drawbacks. First, the absence of qualitative data to explain our results, to compare or relate with, and to ensure completeness may be a limiting factor. Secondly, there is a possibility of social desirability bias because of self-reporting. Thirdly, recall bias is another possibility as the ability to remember past events may depend on various factors. Although this study observed time and distance from facility as access factors, availability and mode of transportation, health facility operating hours, health worker/mother/newborn ratio, and cost of care, impact of weather and payment schemes were not studied. These limitations should be taken into account in the interpretation of our results. In spite of these limitations, our results bring a new standpoint on PNC use in South-Sudan. Our study is the first in Mundri East County to highlight low use of PNC in the first week of delivery (a time when maternal and newborn deaths are high).

Our findings can be used to advocate, design health promotion strategies and messages so as to increase PNC utilization in Mundri East County and other similar settings. In addition, this study sets a benchmark for further PNC research in Mundri East County.

## Conclusions

Overall, the level of EPNC use by postpartum mothers in Mundri East County, Equatorial State is low. Postpartum mothers that accessed health services at government health facilities and took more than an hour to reach the nearest health facility had reduced use of EPNC. Meanwhile, maternal education and PNC health education after delivery were enablers of EPNC use. Our results suggest a pressing need to increase EPNC use among postpartum mothers to avert maternal and newborn deaths within the first week of delivery. Secondly, healthcare providers should improve maternal knowledge of PNC by strengthening PNC health education during ANC visits and before discharge of postpartum mothers from health facilities. In particular, clear messaging should target mothers without and those with low formal education. Thirdly, the Government of South Sudan should improve access to health services (preferably within 5-km radius).

## Abbreviations

ANC: Antenatal care
AOR: Adjusted odds ratio
EPNC: Early postnatal-care
PNC: Postnatal-care
UOR: Unadjusted odds ratio
WES: Western equatorial state
WHO: World Health Organization
